# DISC1-mediated dysregulation of adult hippocampal neurogenesis in rats

**DOI:** 10.3389/fnsys.2015.00093

**Published:** 2015-06-25

**Authors:** Heekyung Lee, Eunchai Kang, Douglas GoodSmith, Do Yeon Yoon, Hongjun Song, James J. Knierim, Guo-li Ming, Kimberly M. Christian

**Affiliations:** ^1^Krieger Mind/Brain Institute, Johns Hopkins UniversityBaltimore, MD, USA; ^2^Institute for Cell Engineering, Johns Hopkins University School of MedicineBaltimore, MD, USA; ^3^Department of Neurology, Johns Hopkins University School of MedicineBaltimore, MD, USA; ^4^The Solomon H. Snyder Department of Neuroscience, Johns Hopkins University School of MedicineBaltimore, MD, USA; ^5^Department of Psychiatry and Behavioral Sciences, Johns Hopkins University School of MedicineBaltimore, MD, USA

**Keywords:** dentate gyrus, adult neurogenesis, hippocampus, DISC1, schizophrenia, psychiatric disorders

## Abstract

Adult hippocampal neurogenesis, the constitutive generation of new granule cells in the dentate gyrus of the mature brain, is a robust model of neural development and its dysregulation has been implicated in the pathogenesis of psychiatric and neurological disorders. Previous studies in mice have shown that altered expression of *Disrupted-In-Schizophrenia 1* (*Disc1*), the mouse homolog of a risk gene for major psychiatric disorders, results in several distinct morphological phenotypes during neuronal development. Although there are advantages to using rats over mice for neurophysiological studies, genetic manipulations have not been widely utilized in rat models. Here, we used a retroviral-mediated approach to knockdown DISC1 expression in dividing cells in the rat dentate gyrus and characterized the morphological development of adult-born granule neurons. Consistent with earlier findings in mice, we show that DISC1 knockdown in adult-born dentate granule cells in rats resulted in accelerated dendritic growth, soma hypertrophy, ectopic dendrites, and mispositioning of new granule cells due to overextended migration. Our study thus demonstrates that the *Disc1* genetic manipulation approach used in prior mouse studies is feasible in rats and that there is a conserved biological function of this gene across species. Extending gene-based studies of adult hippocampal neurogenesis from mice to rats will allow for the development of additional models that may be more amenable to behavioral and *in vivo* electrophysiological investigations. These models, in turn, can generate additional insight into the systems-level mechanisms of how risk genes for complex psychiatric disorders may impact adult neurogenesis and hippocampal function.

## Introduction

Significant progress has been made over the past several years to identify genetic disruptions that increase susceptibility to psychiatric disorders. However, very little is known about how most of these genes contribute to the dysregulation of cellular processes or can influence the integrity of distributed neural systems. One such gene of interest is *Disrupted-In-Schizophrenia 1* (*DISC1*), which was initially identified at the breakpoint of a chromosomal translocation t(1;11)(q42.1;q14.3) that co-segregates with schizophrenia (Millar et al., 2000; Blackwood et al., 2001) and mood disorders (Hamshere et al., 2005; Hashimoto et al., 2006). Unlike most genetic risk factors identified thus far, *Disc1* has been the focus of several investigations into its functional role, and its effect on neuronal development is well-appreciated (Thomson et al., 2013; Wen et al., 2014).

DISC1 is broadly expressed in many brain regions during embryonic development and promotes cell proliferation, migration, and neurite outgrowth (Bradshaw et al., 2011). It also interacts with GSK3beta to regulate neural progenitor proliferation (Kamiya et al., 2005; Shinoda et al., 2007; Mao et al., 2009). In the adult mouse brain, DISC1 expression is more restricted than in the developing brain, with particularly high expression in the dentate gyrus. (Austin et al., 2004). The dentate gyrus is a critical site of adult neurogenesis, the process of generating new dentate granule cells from neural stem cells (Ming and Song, 2011; Braun and Jessberger, 2013). Neural stem cells in the subgranular zone give rise to intermediate neural progenitors and, ultimately, postmitotic newborn neurons that migrate into the inner granule cell layer and become mature dentate granule cells (Altman and Das, 1965; Kaplan and Hinds, 1977; Gage et al., 1998; Lennington et al., 2003; Ming and Song, 2005). Newly generated adult-born neurons establish synaptic connections and functionally integrate into the existing circuitry (van Praag et al., 2002; Ming and Song, 2005; Ge et al., 2006; Rahimi and Claiborne, 2007). Studies have shown that DISC1 is a critical mediator of the tempo of neuronal development and integration during adult neurogenesis in mice (Duan et al., 2007; Kang et al., 2011; Kim et al., 2012a). Strikingly, knockdown of DISC1 in a subset of newborn neurons was sufficient to elicit behavioral impairments (Zhou et al., 2013). Together, these studies suggest that *Disc1* mutations affect not only early neural development but continue to disrupt neuronal development in the hippocampus into adulthood. Dysregulation of adult neurogenesis has been implicated in several psychiatric and neurological disorders, but the causal relevance and potential mechanisms are not well understood (Kitabatake et al., 2007; Christian et al., 2010, 2014).

To investigate risk gene-mediated changes in adult neurogenesis in a model system that is highly amenable to physiological and behavioral experiments, we examined the effects of DISC1 knockdown in neural progenitors in the adult rat hippocampus. Using an oncoretrovirus-mediated RNA interference approach, we characterized the morphological changes of adult-born neurons following DISC1 knockdown. Consistent with results from the studies in mice (Duan et al., 2007; Faulkner et al., 2008; Kim et al., 2009, 2012a; Kang et al., 2011), we show that DISC1 knockdown in adult-born neurons results in soma hypertrophy, accelerated dendritic outgrowth with the appearance of ectopic dendrites, and mispositioning of granule cells due to overextended migration. These findings indicate that DISC1 regulates morphological development and neuronal integration during adult neurogenesis in rats. Further, our study demonstrates the feasibility of utilizing *Disc1* genetic manipulations in rats, providing an alternate animal model to elucidate the functional role of schizophrenia risk genes in adult neurogenesis and hippocampal function.

## Materials and Methods

### Constructs, Production and Stereotaxic Injection of Engineered Oncoretroviruses

Self-inactivating murine oncoretroviruses were engineered to co-express shRNAs under the U6 promoter, and green fluorescent protein (GFP) under the Ubiquitin promoter, to target proliferating cells and their progeny (Kang et al., 2011). Specific shRNAs against *Disc1* (e.g., shRNA-D1) were previously shown to knockdown DISC1 in several mouse lines with rescue experiments to show both efficacy and specificity (Duan et al., 2007; Faulkner et al., 2008; Kim et al., 2009). The D1 hairpin target sequence matches exactly to both mouse and rat *Disc1* sequences (*GGCTACATGAGAAGCACAG*; nucleotide position 17701097–17701115) and its efficacy in rat models was validated in previous studies (Maher and Loturco, 2012). High titers of engineered retroviruses (10^8^ TU/ml) were packaged by Allele Biotechnology (San Diego, CA, USA). Adult male rats (10–12 weeks old; Charles River) were housed under standard 12 h light/dark conditions with *ad libitum* access to food and water. Rats were anesthetized and underwent stereotaxic injections of concentrated retroviruses bilaterally in the hilus/dentate gyrus region at 3 sites per hemisphere (1 μl per site at 0.25μl/min) with the following coordinates: AP = 2.6 mm, ML = ±1.2 mm, DV = 3.8 mm; AP = 3.6 mm, ML = ±2.0 mm, DV = 3.4 mm; AP = 4.6 mm, ML = ±2.8 mm, DV = 3.2 mm. All animal procedures used in this study were performed in accordance with the protocol approved by the Institutional Animal Care and Use Committee at Johns Hopkins University and in accordance with the guidelines of the National Institutes of Health.

### Immunohistology, Confocal Imaging and Analysis

Following perfusion, coronal brain sections (40-μm thick) were prepared from the dissected brains of injected rats at 2 weeks post-injection (wpi) and 4 wpi and processed for immunostaining as previously described (Ge et al., 2006; Duan et al., 2007; Song et al., 2013). Anti-GFP antibodies (goat, 1:500, Rockland) were used in all conditions. Sections were incubated for 30 min in 4′6′-diaminodino-2-phenylindole (DAPI, 1:5000) before washing and mounting. Confocal images were acquired (Zeiss LSM 710) using a multi-track configuration. At least three rats per condition were analyzed. Statistical comparisons of datasets were performed by JMP Statistical Software (SAS).

Morphological analyses were performed using *Z*-series stacks of confocal images. Quantification (NIH ImageJ program) was performed using the confocal image slice that contained the largest soma area for an individual GFP^+^ neuron. Determination of neuronal position was based on single section confocal images of GFP^+^ neurons, counterstained with DAPI, to resolve cell localization among the four areas defined in Figure 3B.

Quantification of dendritic development was based on three dimensional (3D) reconstructions of complete dendritic processes of each GFP^+^ neuron using *Z*-series stacks of confocal images and the 2D projection images were traced with NIH ImageJ. As previously described, every GFP^+^ dentate granule cell that had visibly intact dendritic processes was used in the analysis of total dendritic length and branch number (Ge et al., 2006; Duan et al., 2007; Sun et al., 2013). Corrections for inclinations of dendritic process were not performed and dendritic process reconstructions represent projected lengths.

## Results

**Figure 1:**
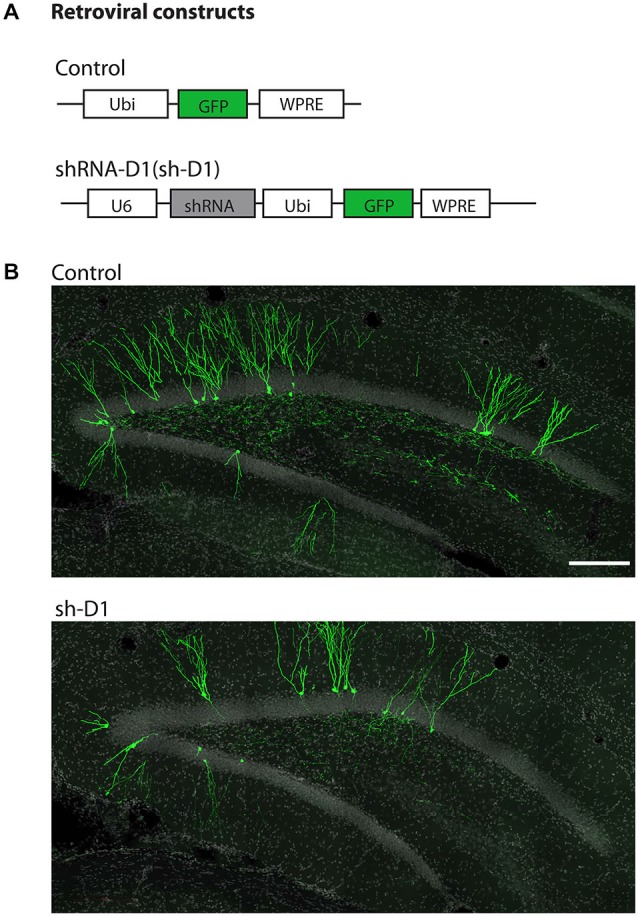
**Stereotaxic injection of retrovirus in the dentate gyrus of the adult rat hippocampus. (A)** Schematic diagram of the retroviral vector used for *in vivo* birth-dating and genetic manipulation. **(B)** Sample projections of *Z*-series confocal images at 4 wpi of retrovirus either expressing GFP (control) or co-expressing shRNA against *Disc1* and GFP (sh-D1) in the dentate gyrus of the adult rat hippocampus. Scale bar: 200 μm.

We used a retrovirus-mediated birth dating and genetic manipulation approach to knock down DISC1 expression in the adult rat dentate gyrus. Engineered retroviral constructs expressed enhanced GFP as control or co-expressed GFP and shRNA to knock down the expression of endogenous rat DISC1 (sh-D1; Figure 1A). Viral constructs were generated as previously described (Kang et al., 2011) and the shRNA sequence used is 100% homologous to the mouse sequence (Duan et al., 2007). High titers of engineered retroviruses were stereotaxically injected into the hilar region of the adult rat hippocampus to selectively target infection of proliferating neural progenitors *in vivo*. GFP^+^ labeled newborn granule cells (green) can be observed in the granule cell layer of the dentate gyrus at 4 wpi of control or sh-D1 retrovirus (Figure 1B). This result demonstrated that the strategy for retroviral-mediated genetic manipulation and labeling newborn neurons in mice was also effective in rats.

### DISC1-Deficient Adult-born Neurons Exhibit Soma Hypertrophy

**Figure 2:**
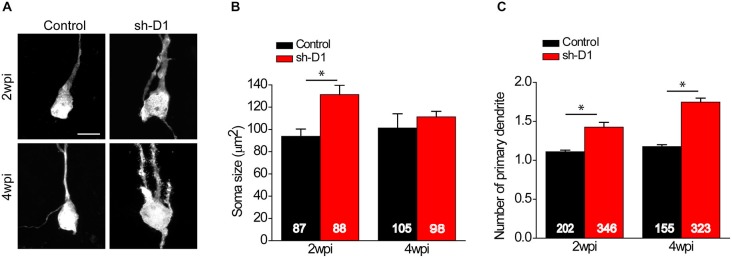
**DISC1 regulates morphogenesis of adult born neurons in the rat hippocampus. (A)** Sample projections of *Z*-series confocal images of GFP^+^ neurons at 2 and 4 wpi of retrovirus expressing GFP (control) or co-expressing shRNA against *Disc1* and GFP (sh-D1). Scale bar: 10 μm. Note that the newborn neurons with DISC1 knockdown show increased soma size and ectopic primary dendrites. **(B)** Quantification of soma size of GFP^+^ neurons at 2 and 4 wpi. Numbers indicated in each bar graph represent the total number of neurons analyzed from 3–8 animals under each condition. Values represent mean ± SEM (*p < 0.05; Two- way ANOVA; 2 wpi: control, *n* = 87, sh-D1, *n* = 88; 4 wpi: control 105, sh-D1, *n* = 98). **(C)** Quantification of number of primary dendrites of GFP^+^ neurons at 2 and 4 wpi. Numbers indicated in each bar graph represent the total number of neurons analyzed from 3–8 animals under each condition. Values represent mean ± SEM (*p < 0.05; Two-way ANOVA; 2 wpi: control, *n* = 202, sh-D1, *n* = 346; 4 wpi: control, *n* = 155, sh-D1, *n* = 323).

We first examined the morphology of sh-D1/GFP^+^ adult-born neurons. The cell bodies of sh-D1/GFP^+^ adult-born neurons were larger than those of control/GFP^+^ neurons (Figure 2A). The mean soma size of the sh-D1/GFP^+^ adult-born neurons was significantly larger than that of the control/GFP+ neurons (Figure 2B; Two-way ANOVA, group: *F*_(1,374)_ = 29.79, *p* < 0.0001; time: *F*_(1,374)_ = 1.26, *p* = 0.26; group × time: *F*_(1,374)_ = 10.78, *p* = 0.0011). The soma size of the sh-D1/GFP^+^ neurons was significantly different from the control/GFP^+^ neurons at 2 wpi (*post hoc* Tukey HSD, *p* < 0.05). A similar transient phenotype of enlarged soma size was also observed in developing human cortical neurons derived from patient induced pluripotent stem cells carrying a *DISC1* mutation (Wen et al., 2014). Dentate granule cells in rodents normally extend only one primary apical dendrite, which branches out to form an elaborate arborization (Seress and Pokorny, 1981; Shapiro and Ribak, 2006). However, neurons with DISC1 knockdown exhibited ectopic primary dendrites (Figure 2C). The number of primary dendrites in the sh-D1/GFP^+^ neurons was significantly greater than the control/GFP^+^ neurons at both 2 and 4 wpi (Two-way ANOVA, group: *F*_(1,795)_ = 128.68, *p* < 0.0001; time: *F*_(1,795)_ = 25.20, *p* < 0.0001; group × time: *F*_(1,795)_ = 7.71, *p* = 0.0056; *Post hoc* Tukey HSD, *p* < 0.05). These results showed that DISC1 regulates the morphogenesis of adult-born neurons in rats.

### DISC1 Knockdown Cells Exhibit Aberrant Positioning in the Dentate Gyrus

**Figure 3:**
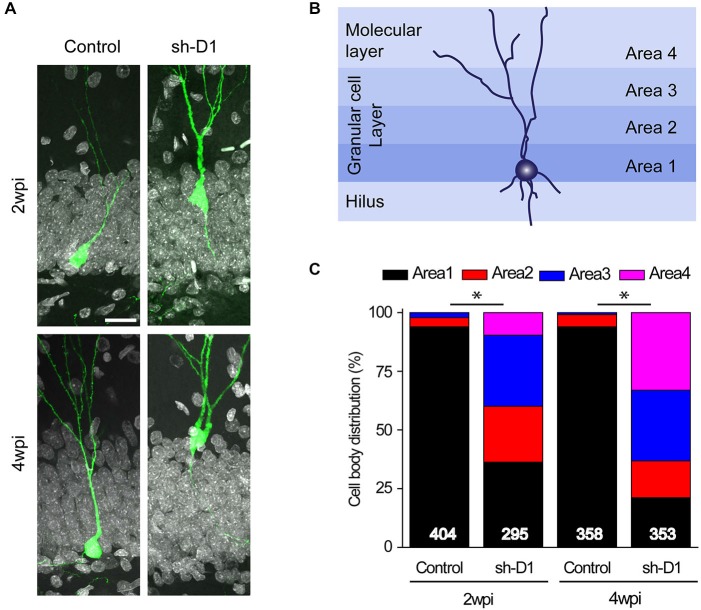
**DISC1 regulates the positioning of the adult born neurons in the rat hippocampus. (A)** Sample confocal images of GFP and DAPI. Scale bar: 20 μm. **(B)** Schematic diagram of the adult rat dentate gyrus region divided into four domains. **(C)** Distribution of GFP^+^ cells within each area as defined in **(B)**. Numbers indicated in each bar graph represent the total number of neurons analyzed from 3–8 animals under each condition (2 wpi: control, *n* = 404, sh-D1, *n* = 295; 4 wpi: control, *n* = 358, sh-D1, *n* = 353).

Next, we examined whether knockdown of DISC1 affected the migration of adult-born neurons. Adult-born neurons in the dentate gyrus contribute almost exclusively to only the inner two-thirds of the granule cell layer (Areas 1 and 2; Figure 3B; Kempermann et al., 2003). However, the distributions of the control/GFP^+^ and the sh-D1/GFP^+^ neurons in the granule and the molecular cell layers were significantly different (Figure 3C: Three-way Chi Square Test, *G*^2^ = 885.2, *p* < 0.0001). Most of the control/GFP^+^ neurons migrated into the inner layer of the granule cell layer (Area 1) at 2 and 4 wpi (Figures 3A,C). In contrast, at 2 wpi, the sh-D1/GFP^+^ neurons had already migrated into the middle (Area 2) and outer third (Area 3) of the granule cell layer with some cells even migrating into the molecular layer (Area 4; Figures 3A,C). By 4 wpi, the majority of the sh-D1/GFP^+^ neurons were in the outer third of the granule cell layer (Area 3) and the molecular layer (Area 4), while none of the control/GFP^+^ neurons were in the molecular layer (Area 4; Figures 3A,C). These results demonstrated that DISC1 regulates the positioning of adult-born neurons in rats.

### DISC1 Modulates Dendritic Development of Adult-Born Neurons

**Figure 4:**
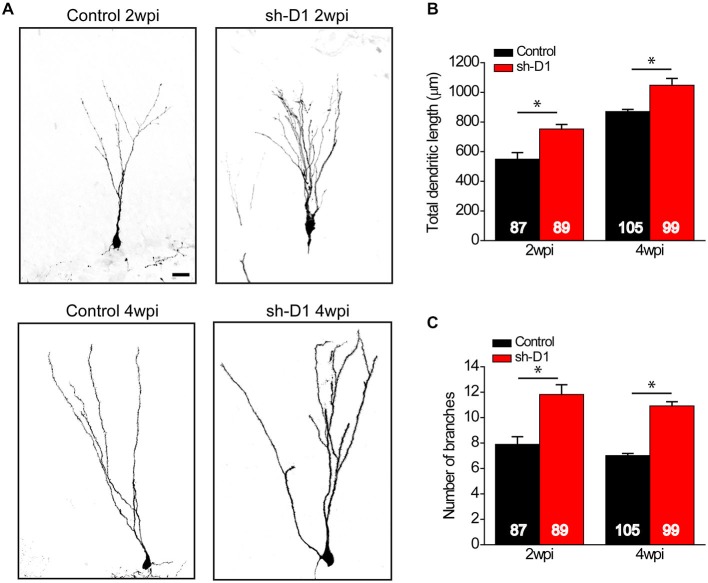
**DISC1 regulates the dendritic development of adult born neurons in the rat hippocampus. (A)** Sample projections of *Z*-series confocal images of GFP^+^ neurons at 2 and 4 wpi. Scale bar: 10 μm. **(B)** Total dendritic length of GFP^+^ neurons at 2 and 4 wpi. Numbers indicated in each bar graph represent the total number of neurons analyzed from 3–8 animals under each condition. Values represent mean ± SEM (*p < 0.05; Two-way ANOVA; 2 wpi: control, *n* = 87, sh-D1, *n* = 89; 4 wpi: control, *n* = 105, sh-D1, *n* = 99). **(C)** Number of branches of GFP^+^ neurons at 2 and 4 wpi. Values represent mean ± SEM (*p < 0.05; Two-way ANOVA; 2 wpi: control, *n* = 87, sh-D1, *n* = 89; 4 wpi: control, *n* = 105, sh-D1, *n* = 99).

We also examined the effect of DISC1 knockdown on the dendritic development of the adult-born neurons. Sh-D1/GFP^+^ neurons exhibited much more elaborate dendrites than the control/GFP^+^ neurons both at 2 and 4 wpi (Figure 4A). Total dendritic length (Figure 4B: Two-way ANOVA, group: *F*_(1,376)_ = 50.18, *p* < 0.0001; time: *F*_(1,376)_ = 117.87, *p* < 0.0001; group × time: *F*_(1,376)_ = 0.28, *p* = 0.59) and the number of dendrite branches (Figure 4C: Two-way ANOVA, group: *F*_(1,376)_ = 112.39, *p* < 0.0001; time: *F*_(1,376)_ = 4.64, *p* = 0.03; group × time: *F*_(1,376)_ = 0.09, *p* = 0.77) were significantly greater for the sh-D1/GFP^+^ neurons at both 2 and 4 wpi than the control/GFP^+^ neurons (*Post hoc* Tukey HSD, *p* < 0.05). Thus, DISC1 regulates the dendritic development of adult-born neurons in rats.

## Discussion

We have shown that knockdown of DISC1, caused abnormal morphological changes in adult-born dentate granule cells in rats. DISC1 knockdown resulted in soma hypertrophy, accelerated dendritic outgrowth with the appearance of ectopic dendrites, and mispositioning of new granule cells due to overextended migration. These findings are consistent with previous findings in mice (Duan et al., 2007) showing that DISC1 also orchestrates the tempo of neuronal integration during adult neurogenesis in rats. Our results support a conserved biological function of DISC1 in rats and mice and the viability of a viral-mediated approach to manipulate risk genes in adult hippocampal neurogenesis in rats.

Determining the biological role of psychiatric disorder risk genes in neuronal development, structure, and function is a critical step toward understanding how these genes may contribute to dysregulation of neural processes necessary for adaptive behavior. Many risk genes have been identified but only a few have been studied in detail, most of which have been investigated exclusively in mice. And the majority of these studies show a correlation between genetic risk variants and changes in behavior and/or cellular properties, but very few have been focused on identifying mechanisms at the systems level. Although mice are highly amenable to genetic manipulation, there are a wealth of behavioral data in other species and several advantages in using rats. First, rat models have been used extensively in physiological studies and provide technical advantages. The size of the rat allows for larger implants that can support more tetrodes for unit recordings, compared to what can be implanted in mice. A greater number of tetrodes for recording results in a larger number of cells that can be recorded simultaneously to study network ensemble activities. Second, studies have shown that place fields can be less stable in mice than in rats (Kentros et al., 2004; Muzzio et al., 2009). Place cells in the hippocampus are thought to contribute to pattern separation by reorganizing their spatially-tuned firing patterns, referred to as “remapping”, which is induced when there are changes to the input patterns (Muller and Kubie, 1987; Bostock et al., 1991; Leutgeb et al., 2005, 2007; Neunuebel and Knierim, 2014). Although place field instability in mice is not well understood, it may cause difficulties in interpreting and understanding hippocampal place cell properties and functions related to remapping. Third, studies have shown that a higher percentage of mature neurons survive in rats as compared to mice (Snyder et al., 2009). Thus, to study the physiological effects of DISC1 knockdown in newborn neurons, rat models provide an important complement to existing mouse models.

Evidence suggests that adult hippocampal neurogenesis itself may be important in psychiatric disorders, including schizophrenia (Toro and Deakin, 2007; Eisch et al., 2008; Kempermann et al., 2008; DeCarolis and Eisch, 2010). Neuroimaging and postmortem neuropathologic studies indicate decreased hippocampal volume and function in patients with schizophrenia (Lawrie and Abukmeil, 1998; Nelson et al., 1998; Mccarley et al., 1999; Wright et al., 2000; Goldman and Mitchell, 2004; Harrison, 2004). One study showed a reduction in putative precursor cell proliferation in schizophrenia patients, suggesting a direct link between adult neurogenesis and schizophrenia (Reif et al., 2006). Moreover, animal models of schizophrenia exhibit altered adult neurogenesis, and antipsychotic treatments can normalize the changes (Liu et al., 2006; Keilhoff et al., 2010; Procaccini et al., 2011; Wolf et al., 2011). Despite these putative associations between dysregulation of adult neurogenesis and schizophrenia, there are little data to support a causal role of aberrant neurogenesis in the emergence or maintenance of relevant symptomatology.

Schizophrenia is a developmental disorder and thus adult neurogenesis may be most informative as a model system in which to explore how genetic risk factors may lead to specific molecular, cellular and circuit-level phenotypes (Singh et al., 2004). In the patient population, consequences of genetically-mediated risk could be present in any region of the brain and cell type in which the gene is normally expressed. In contrast, our manipulation affects a specific cell type in the dentate gyrus and it is difficult to ascribe any subset of the diverse and complex array of clinical symptoms to impairments in this region alone. Nevertheless, the hippocampus is a critical site of learning and memory and the dentate gyrus appears to play a role in several adaptive behaviors that could directly or indirectly contribute to some of the core symptomatology. Among the specific behaviors that have been associated with the dentate gyrus and/or neurogenesis are spatial learning, contextual and spatial discrimination, temporal encoding, associative learning, and anxiety and mood regulation (Valenzuela-Harrington et al., 2007; Procaccini et al., 2011; Sahay et al., 2011; Zhou et al., 2013; Carretero-Guillén et al., 2015), which have been reviewed in detail elsewhere (Kim et al., 2012c; Aimone et al., 2014; Miller and Hen, 2015). Although both upregulation and downregulation of adult neurogenesis can result in clear behavioral phenotypes, dysregulation of this process can take many different forms and could have distinct effects on the local circuitry and information processing in this region.

In order to understand how adult neurogenesis contributes to hippocampal function and, conversely, how its genetically-mediated dysregulation can lead to impairments that may be associated with psychiatric or neurological disorders, we need to be able to test explicit hypotheses in a well-established system.

Both as a model for neural development and an intrinsic phenomenon with a functional role in behaviors mediated by the hippocampus, adult neurogenesis is a focus of many studies. By demonstrating that manipulation of a psychiatric disorder risk gene has a clear effect on neuronal development in the adult rat hippocampus, we have established another viable model in which to investigate the physiological correlates of aberrant neurogenesis in the hippocampus.

## Author Contributions

HL collected data, EK analyzed data, DG helped collect data, DY helped analyze data, HS, JJK, G-lM, and KMC designed and organized the research. HL, EK, and KMC wrote the manuscript. All authors contributed in discussion of the results and the manuscript.

## Conflict of Interest Statement

The authors declare that the research was conducted in the absence of any commercial or financial relationships that could be construed as a potential conflict of interest.
